# Social Determinants of Health in Digital Health Policies: an International Environmental Scan

**DOI:** 10.1055/s-0044-1800759

**Published:** 2025-04-08

**Authors:** Jiyoun Song, Mollie Hobensack, Lydia Sequeira, Hwayeon Danielle Shin, Shauna Davies, Laura-Maria Peltonen, Dari Alhuwail, Nader Alnomasy, Lorraine J. Block, Sena Chae, Hwayoung Cho, Hanna von Gerich, Jisan Lee, James Mitchell, Irem Ozbay, Erika Lozada-Perezmitre, Charlene Esteban Ronquillo, Sang Bin You, Maxim Topaz

**Affiliations:** 1 University of Pennsylvania School of Nursing, Philadelphia, Pennsylvania, United States; 2 Icahn School of Medicine at Mount Sinai, Department of Geriatrics and Palliative Care, New York, United States; 3 Centre for Addiction and Mental Health, Toronto, Ontario, Canada; 4 Institute of Health Policy, University of Toronto, Toronto, Ontario, Canada; 5 University of Regina, Faculty of Nursing, Regina, Saskatchewan, Canada; 6 Department of Nursing Science, University of Turku and Research Services, Turku, Finland; 7 Information Science Department, College of Life Sciences, Kuwait University, Kuwait City, Kuwait; 8 University of Hail, College of Nursing, Hail, Saudi Arabia; 9 University of British Columbia, School of Nursing, Vancouver, British Columbia, Canada; 10 University of Iowa, College of Nursing, Iowa City, Iowa, United States; 11 University of Florida, College of Nursing, Gainesville, Florida, United States; 12 Gangneung-Wonju National University, Department of Nursing, Wonju, Republic of Korea; 13 University of Colorado School of Medicine, Department of Biomedical Informatics, Colorado, United States; 14 Istanbul Sabahattin Zaim University, Faculty of Health Sciences, Department of Nursing, Istanbul, Türkiye; 15 Faculty of Nursing, Benemerita Universidad Autonoma de Puebla, Nursing Faculty BUAP, Puebla, México; 16 University of British Columbia, School of Nursing, Okanagan, Kelowna, British Columbia, Canada; 17 University of Pennsylvania School of Nursing, Department of Biobehavioral Health Sciences, Philadelphia, Pennsylvania, United States; 18 Columbia University School of Nursing & VNS Health, New York City, New York, United States

**Keywords:** Social Determinant of Health, Digital Health, Health Policy, Environmental Scan, Nursing Informatics

## Abstract

**Introduction** : Social Determinants of Health (SDoH) include factors such as economic stability, education, social and community context, healthcare access, and the physical environment, which shape an individual's health and well-being. Given that the inclusion of SDoH factors is essential in improving the quality and equity of digital health, this study aims to examine how SDoH is incorporated within digital health policies internationally.

**Methods** : An environmental scan of digital health policies was conducted, including relevant documents from multiple countries and global organizations. Key content related to SDoH was extracted from the documents, and a content analysis was conducted to identify seven different SDoH domains (i.e., target audience, SDoH inclusion, addressing health inequities, SDoH-related key performance indicators, data collection on SDoH, interoperability standards, and data privacy and security). Data were aggregated at the global and continental levels to integrate and synthesize information from different countries and regions.

**Results** : A total of 28 digital health policies or strategies were identified across 16 international regions. The comparative analysis of health policies regarding SDoH reveals a pronounced disparity between the continental regions. Although the World Health Organization recognizes the significance of key performance indicators for monitoring SDoH and emphasizes the assessment of national digital health maturity, there's a noticeable lack of continent-specific policies reflecting these global initiatives at the continental level.

**Conclusion** : While some regional digital health strategies recognize SDoH, integration varies, and standardization is lacking. Future research should focus on data collection frameworks and comprehensive insights for policymakers.

## 1. Introduction

Health informatics and digital health are a convergence of healthcare and technology that optimize patient care, streamline processes, and enhance health outcomes [ 1 , 2 ]. At its core, digital health involves the application of information technology to efficiently manage, analyze, and utilize healthcare data, including the critical aspect of health information systems for data management and exchange [ 3 4 5 ]. In addition to data management, digital health encompasses telemedicine, telehealth, and remote monitoring solutions [ 6 ]. Telemedicine and telehealth allow remote access to healthcare services, overcoming geographic barriers, and remote monitoring provides real-time data for timely interventions. This context emphasizes the significance of digital health policies, which establish a regulatory framework governing the utilization of digital health technologies, safeguarding patient data, and upholding ethical, equitable, and secure practices. The development of digital health policies is instrumental in maintaining data privacy, protecting against cyber-attacks, and establishing standards for data interoperability across healthcare systems [ 7 8 9 ]. In addition to promoting innovation and standardization, digital health policies can provide guidance and incentives for the development of cutting-edge healthcare technologies and solutions [ 10 ]. As a result, this initiative fosters a culture of research and development, encouraging the continuous improvement of digital health tools to meet changing healthcare requirements.

The impact of digital health and its associated policies on healthcare service delivery, accessibility, and outcomes underscores the critical need for policies prioritizing equity within the framework of digital health. Recent studies have emphasized the vital role of digital health in integrating measures to enhance healthcare access and address disparities, highlighting their interconnection with healthcare delivery and policymaking [ 11 , 12 ]. The purpose of these policies is not only to safeguard patient data, but also to ensure ethical, equitable, and secure practices, enabling us to address health disparities more effectively. In accordance with these movements, the International Council of Nurses (ICN) and the National Institute of Nursing Research (NINR) have urged governments and institutions to implement substantial, effective, and enduring policy reforms to achieve health equity and reduce disparities [ 13 , 14 ]. A key component of these reforms is to comprehensively incorporate the consideration of social determinants of health (SDoH) into healthcare policies and practices. SDoH encompasses the multifaceted non-medical factors that profoundly shape an individual's health and well-being, including economic stability, education, social and community context, healthcare access, and the physical environment [ 15 , 16 ]. It has become increasingly recognized that SDoH impacts health outcomes and health disparities [ 17 18 19 ].

For instance, an individual's economic status can directly impact health by affecting access to essential resources such as nutritious food, safe housing, and education. Education also plays a significant role in health literacy and informed decision-making. Social and community factors, including support networks and available resources, can either mitigate or exacerbate stressors and health risks. The physical environment, encompassing air and water quality, housing conditions, and neighborhood safety, directly and indirectly impacts health. Furthermore, healthcare access, including affordability, availability, and cultural competence, can significantly influence health outcomes. Insufficient access to healthcare can lead to delayed diagnosis and treatment, negatively affecting overall health. Moreover, the digital divide should be considered considering the current healthcare landscape and ongoing discussions regarding digital health policies in the transition to healthcare digitization [ 20 , 21 ]. The digital divide is intricately linked to and shaped by SDoH, such as access to technology and connectivity, proficiency in utilizing these technologies effectively, and the ability to translate technology usage into practical results [ 22 ]. To prevent the exacerbation of the digital divide and mitigate potential unintended consequences, recognizing the significance of SDoH becomes essential in formulating comprehensive digital healthcare strategies.

A comprehensive environmental scan provides policymakers with valuable insights into the extent to which these determinants are considered in digital health policies worldwide [ 23 ]. In 2015, a study conducted by researchers and policymakers in the United States examined national health-focused metrics to evaluate the extent to which these initiatives explicitly considered social, environmental, and economic health determinants within the country [ 24 ]. However, none of the existing studies had explicitly addressed the incorporation of SDoH into international digital health policies. To address this knowledge gap, this effort aims to enable international collaboration and enhance global healthcare standards, with a special focus on SDoH in the digital health environment.

This study aims to examine how SDoH is incorporated into digital health policies internationally, encompassing approaches and policies from different countries, and to provide recommendations for the inclusion of fundamental perspectives of SDoH in digital health policy development.

## 2. Method

### 2.1. Search Strategy

Using a comprehensive search strategy, we conducted an international environmental scan of digital health policies and their relationship to SDoH. The purpose of this strategy was to capture relevant documents, reports, and publications both from global sources and country-specific sources. To ensure a diverse and comprehensive dataset, we followed a structured approach throughout our search process, which was conducted on December 28, 2023.

(1) *Platform Selection :* We identified and selected appropriate online platforms representing different regions and countries. These platforms included government websites, health department portals, official digital health strategy pages, and websites of relevant international health organizations.

(2) *Search Query Formulation* : Keyword searches were utilized to enhance the data collection process. An extensive set of keywords covering different aspects of digital health policies and their correlation with social determinants of health was devised. The queries were designed to retrieve documents, reports, and publications related to digital health policies. These keywords included terms such as “digital health policies”, “digital health strategy”, “health informatics policies”, “health informatics strategy”, “social determinants of health”, and specific country or region names. Boolean operators (AND, OR) were effectively used to combine keywords and refine search results.

(3) Geographic Scope: In our research, we delineated the geographical scope for each region or country, clarifying whether our search encompassed the entire nation, specific states or provinces, or regional health authorities. Within the framework of international research groups ( https://imia-medinfo.org/wp/ ), co-authors from various countries were tasked with conducting searches and gathering pertinent policy documents and reports within their respective regions.

### 2.2. Inclusion and Exclusion Criteria

We incorporated documents and publications directly connected to digital health policies (“e-health” or “health informatics”, as applicable in some countries), digital health interventions, and their potential influence on SDoH. These documents encompassed policy reports, academic articles, government guidelines, and official statements. Additionally, we excluded sources unrelated to the topic, not available in English (with some allowances for documents from certain countries where one of the co-authors could translate), or outdated (published before the year 2020). Our exclusion criterion was based on the consideration that policy strategies are developed on a five- or ten-year cycle, making recent sources more relevant.

### 2.3. Data Extraction

A comprehensive literature review was conducted to identify relevant frameworks, guidelines, and prior studies regarding assessing SDoH in healthcare policies and digital health strategies to develop our data extraction framework [ 25 26 27 28 29 30 31 ]. With this extensive review as a foundation, we adapted and refined existing frameworks [ 32 33 34 ] while introducing elements unique to digital health. This iterative process involved consultations with subject-matter experts and researchers specializing in health policy analysis (JS, LS, and HDS). The data extraction instrument was designed to align with recognized frameworks for SDoH assessment as well as to investigate the unique nuances of digital health, including considerations related to interoperability, data privacy, and the utilization of technology to bridge health disparities. It served as a standardized and comprehensive framework, ensuring that no critical aspect of SDoH within digital health policies was overlooked during data collection. Our data extraction instrument enabled cross-regional and cross-country comparisons and ensured the scientific rigor of the data extraction process.

We, particularly experts in health informatics internationally, independently identified and acquired official health policy and strategy documents from government and health organization websites, methodically annotating them to indicate the presence or absence of relevant content with additional comprehensive descriptions and details. Given the diverse linguistic landscape of digital health policies and strategies across different regions and countries, our extraction team included members proficient in the languages relevant to the documents under review. Cross-verification by two team members (JS and MH with doctorate degrees and extensive experience in health informatics research) ensured that the extracted data were reliable. Discrepancies were resolved through team discussions.

### 2.4. Data Analysis

The collected data underwent qualitative analysis employing a content analysis approach [ 35 ] that utilized multiple frameworks to categorize the extracted narrative data and discern predominant patterns within digital health policies across various countries. This analysis encompassed categorizing elements such as target audience, SDoH inclusion, addressing health inequities, SDoH-related key performance indicators, data collection on SDoH, interoperability standards, and data privacy and security. Subsequently, the data was aggregated at the global level and the level of the continental regions to integrate and synthesize information from different regions and countries to provide a comprehensive overview. These continental regions included Africa, Asia, America (North), America (South), Europe, and Oceania. To consolidate the information, when a specific item was found in the policies of each country and its prevalence exceeded 75% at the continental level, it was categorized as being present at the continental level. Then, digital health policy approaches were compared across continents to highlight the similarities and differences.

## 3. Results

Our global research examined digital health policies and strategies from 16 regions worldwide and a total of 28 online resources were included for analysis (see Table 1 ). The results included one global digital health initiative developed by the World Health Organization (WHO). The African continent was comprehensively covered, with policies from the African Union, as well as a specific focus on East Africa, totaling two policies. In Asia, our analysis encompassed one policy each from Saudi Arabia and Japan, and two from Korea, summing up to four policies. A total of five policies were analyzed within North America, including two from the United States and three from Canada. In South America, one policy was represented by Mexico. Europe presented a rich variety of policies, totaling 10 in number. These policies included initiatives from the European Commission, three from the United Kingdom, one from Spain, one from Sweden, one from Norway, and three from Finland. Lastly, Oceania (Australia) contributed five.

**Table 1.** List of included online resources for examining digital health policy/strategies for analysis.

Continent Regional Level (Countries)	Organization Name	The official name of the digital health policy/strategy	Year of Initiative	Link to strategy
Global Level	World Health Organization (WHO)	WHO: Global strategy on digital health 2020-2025	2020-2025	https://www.who.int/docs/default-source/documents/gs4dhdaa2a9f352b0445bafbc79ca799dce4d.pdf
Africa: Africa Union (Central, Eastern, Northern, Southern and Western Africa)	The African Centres for Disease Control	Digital Transformation Strategy	From 2022	https://africacdc.org/download/digital-transformation-strategy/
Africa (East Africa)	Government of the Republic of Malawi Ministry of Health	National Digital Health Strategy 2020-2025	2020-2025	https://www.healthdatacollaborative.org/fileadmin/uploads/hdc/Documents/Country_documents/Malawi/Malawi_Digital_Health_Strategy__20-25.pdf
Asia (Saudi Arabia)	The Ministry of Health	National e-Health Strategy	2020-2030	https://www.moh.gov.sa/en/Ministry/nehs/Pages/Ehealth.aspx
Asia (Korea)	Ministry of health and welfare	2023 Work plan	From 2023	https://www.mohw.go.kr/menu.es?mid=a10701000000
	Ministry of health and welfare	Strategy for Creating a New Biohealth Market	From 2023	https://www.president.go.kr/download/64054af013d01
Asia (Japan)	Headquarters for Healthcare Policy of Japan	Global Health Strategy of Japan	2022-2023	https://www.kantei.go.jp/jp/singi/kenkouiryou/en/pdf/final_GHS.pdf
America: North (United States)	United States Agency for International Development	USAID: A vision for action in digital health	2020-2024	https://www.usaid.gov/sites/default/files/2022-05/USAID-A-Digital-Health-Vision-for-Action-v10.28_FINAL_508.pdf
	White House: Domestic Policy Council Office of Science and Technology Policy	The US playbook to address social determinants of health	By 2030	https://www.whitehouse.gov/wp-content/uploads/2023/11/SDOH-Playbook-3.pdf?utm_campaign=Washington%20Download&utm_medium=email&_hsmi=283043455&_hsenc=p2ANqtz-9EGtmSfBmqfbNbS6yqYf4I65ADiN-k4fmAGXHgMNbvbd4lyLI8FqnDRd9cbEzoC07dfO0QOqEum22CHvOFtCBJylckGQ&utm_content=283043455&utm_source=hs_email
America: North (Canada)	Pan-Canadian Health Data Strategy Expert Advisory Group	Digital Health Canada's “Canadian Digital Healthcare Strategies: Insights on Common Themes, Priorities, and Calls to Action”/ Digital Health Canada's Strategy map	From 2023	https://digitalhealthcanada.com/membership/chief-executive-forum/inititatives/strategy-working-group/ ; https://digitalhealthcanada.com/wp-content/uploads/2023/10/CHIEF-Strategy-Working-Group-Summary-v100223.pdf
	Pan-Canadian Health Data Strategy Expert Advisory Group	Canadian Health Data Strategy (Expert Advisory Group Reports)	From 2020	https://www.canada.ca/en/public-health/corporate/mandate/about-agency/external-advisory-bodies/list/pan-canadian-health-data-strategy-reports-summaries/expert-advisory-group-report-03-toward-world-class-health-data-system.html
	Ontario Health's Communications Department	Digital Standards in Health Care	From 2022	https://www.ontariohealth.ca/system-planning/digital-standards
America: South (Mexico)	Organization for Economic Cooperation and Development (OECD)	Digital Government in Mexico	From 2020	https://www.oecd-ilibrary.org/governance/digital-government-in-mexico_6db24495-en
Europe	European Commission	A European Strategy for data: European Health Data Space	From 2022	https://health.ec.europa.eu/ehealth-digital-health-and-care/european-health-data-space_en https://digital-strategy.ec.europa.eu/en/policies/strategy-data
Europe (Finland)	Ministry of Social Affairs and Health	Strategy for digitalisation and information management in healthcare and social welfare	2023–2035	https://julkaisut.valtioneuvosto.fi/handle/10024/165288
	The foundation of Finnish national archive of health information	The national KANTA Health Archive Roadmap 2024-2027	2024-2027	https://www.kanta.fi/ammattilaiset/painopisteet
	Finnish institute for health and welfare	E-health and e-welfare of Finland : Check Point 2022	2020-2021	https://www.julkari.fi/handle/10024/145973
Europe (Norway)	National Council for eHealth	National eHealth strategy	2023-2030	https://www.ehelse.no/strategi/nasjonal-e-helsestrategi-for-helse-og-omsorgssektoren/_/attachment/inline/69d58858-d324-48fe-8d2f-998cda199367:9a2712f7606599c7909bb182a00f1925ba3544e1/National%20eHealth%20strategy%20EN%20MASTER%20v%201.0.pdf
Europe (Spain)	National Health System	Digital health strategy	2021-2026	https://www.sanidad.gob.es/ciudadanos/pdf/Digital_Health_Strategy.pdf
Europe (Sweden)	Government by Ministry of Health and Social Affairs	A strategy for implementing vision for eHealth 2025: The next step	2020-2022	https://ehalsa2025.se/wp-content/uploads/2021/02/Strategy-2020-2022_eng.pdf
Europe (United Kingdom)	United Kingdom: Department of Health & Social Care	United Kingdom: A plan for digital health and social care	2020-2025	https://www.gov.uk/government/publications/a-plan-for-digital-health-and-social-care/a-plan-for-digital-health-and-social-care
	The National Health Service (NHS)	Digital Health and Care Strategy	2021-2026	https://www.oxfordhealth.nhs.uk/wp-content/uploads/2021/11/Oxford-Health-Digital-Health-Care-Strategy.pdf
	Northern Ireland Department of Health	Digital Strategy Health and Social Care Northern Ireland 2022-2030	2022-2030	https://www.health-ni.gov.uk/sites/default/files/publications/health/doh-hscni-digital-strategy-final.pdf
Oceania (Australia)	Australian Digital Health Agency	Australia's National Digital Health Strategy	By 2022	https://www.digitalhealth.gov.au/about-us/strategies-and-plans/national-digital-health-strategy-and-framework-for-action
	Government of Western Australia Department of Health	WA Health Digital Strategy 2020–2030	2020-2030	https://www.health.wa.gov.au/~/media/Files/Corporate/Reports-and-publications/Digital-strategy/Digital-Strategy-2020-2030.pdf
	Western Victoria Primary Health Network	Digital Health Strategy 2023-2026	2023-2026	https://westvicphn.com.au/wp-content/uploads/2023/02/WVPHN0028_DigitalHealthStrategy_v4.pdf
	The Department of Health Tasmania	Digital Health Transformation – Improving Patient Outcomes 2022-2032	2022-2032	https://www.health.tas.gov.au/sites/default/files/2022-06/Digital%20Health%20Transformation%20Improving%20Patient%20Outcomes_0.pdf
	Sydney Local Health District	Digital Health Strategy	2022-2027	https://www.slhd.nsw.gov.au/pdfs/Digital-Health-Strategy2022-27.pdf

### 3.1. A summary of the specific domains covered under each policy

Table 2 shows the summary of specific domains covered under each policy by continental regions. The policy of the WHO is primarily centered on digital health providers and consumers at the primary healthcare level. Conversely, other health policies and strategies typically have a broader scope, aiming to address the needs of general population groups or demographics rather than targeting specific population subsets. The global, Asia, and North American policies specifically mentioned vulnerable or underserved populations including geography, race, ethnicity, age, income or disability.

**Table 2.** Evaluation of continent-wide policies based on domains.

Domain	Item	Continental regions						
		Global Level	Africa	Asia	America (North)	America (South)	Europe	Oceania
1. Target Audience	1.1. Are specific population groups or demographics mentioned as focus areas?	O	X	X	X	X	X	X
	1.2. Does the policy/strategies consider vulnerable or underserved populations?	O	X	O	O	X	X	X
2. SDoH Inclusion	2.1. Does the policy explicitly mention social determinants of health (SDoH) as factors that affect health outcomes?	O	X	X	X	O	X	X
	2.2. Does it provide definitions or explanations of SDOH?	O	X	X	X	O	X	X
	2.3. Are there examples of how SDoH are considered in healthcare strategies?	O	X	O	O	O	X	X
3. Addressing Health Inequities	3.1 Does the policy outline strategies to reduce health disparities and inequities among different population groups?	O	X	O	O	O	X	X
	3.2. Are there specific goals or targets related to equity?	O	X	X	X	O	X	X
	3.3. Does it mention actions to address the root causes of health disparities?	O	X	X	X	X	X	X
	3.4. Does it include digital strategies to alleviate the digital divide?	O	X	O	X	O	X	O
4. SDoH-Related Key Performance Indicators	4.1. Are specific key performance indicators related to social determinants of health defined within the policy to measure progress?	O	X	X	X	X	X	X
	4.2. Are there targets or benchmarks associated with these key performance indicators?	O	X	X	X	X	X	X
5. Data Collection on SDoH	5.1. Does the policy mandate collecting and analyzing data on social determinants of health to assess their impact on healthcare outcomes?	X	X	X	X	X	X	X
	5.2. What types of data on SDoH are to be collected?	X	X	X	X	X	X	X
	5.3. Are data sources and methodologies specified?	X	X	X	X	X	X	X
6. Interoperability Standards	6.1. Does the policy emphasize the importance of interoperability standards for data sharing among healthcare systems?	O	O	X	O	O	O	O
	6.2. Are specific interoperability standards mentioned?	X	O	O	X	O	X	X
	6.3. Does it address cross-border data sharing, if applicable?	O	O	O	X	O	O	X
7. Data Privacy and Security	7.1. Are there clear provisions in the policy addressing data privacy, security, and patient consent in digital health initiatives?	O	O	O	O	O	O	X
	7.2. Does it mention compliance with data protection regulations?	O	O	O	O	O	O	X
	7.3. Are there mechanisms for reporting data breaches or incidents?	X	X	X	X	O	X	X

Note: In the table, the symbol “O” signifies the presence of the evaluated item as stipulated within the designated domain in the established strategy/policy, whereas the symbol “X” denotes the absence of this condition.

The comparative analysis of health policies regarding SDoH reveals a pronounced disparity between the continental regions. While the Global Level, Asia, and the Americas proactively acknowledge and integrate SDoH into their health policies, other continents are not clearly demonstrating this. South America notably stands out in explicitly mentioning SDoH, offering definitions, and setting specific goals related to equity, indicating a strategic and comprehensive approach to health policy. However, continents such as Africa, Europe, and Oceania have been left with unclear SDoH related explanations, concrete examples, and strategic objectives in their digital health policy. In addition, despite the WHO's acknowledgment of the importance of key performance indicators for tracking SDoH and its emphasis on evaluating national digital health maturity, which encompasses essential elements such as digital health infrastructure, knowledge, technologies, and usage, there was a notable absence of consideration for global initiatives at the continental level, with no continent-specific policy reflecting these aspects. Also, no strategy explicitly mentions the SDoH or provides details about the data sources in its policy.

According to the policies included in this study, interoperability standards are widely recognized at the global level and on all continents. The majority of continents, with the exception of North America and Oceania, acknowledge the importance of cross-border data sharing. Except for Oceania, most continents explicitly address privacy, security, consent, and data protection regulations as part of their digital health initiatives.

## 4. Discussion

This study examined how SDoH is incorporated within digital health policies internationally. Our results highlight the importance of each government honing their efforts to integrate and solidify SDoH into their digital health policies to address health inequities in alignment with global initiatives. To enhance public policymaking, clear operational definitions of key terms and concepts are required to ensure the establishment of objectives, targets, and priorities and to assess progress accordingly [ 9 , 16 ]. There are some positive indicators, such as the recognition of SDoH in healthcare strategies at the global level organization and widespread regional acceptance of interoperability standards and data privacy and security. This broad acknowledgment underscores the global commitment to enhancing healthcare data exchange, ensuring the secure transfer of information across borders, and safeguarding patient data in digital healthcare systems [ 36 ]. Nevertheless, further efforts should be made to establish mechanisms for reporting data breaches or incidents, which is of critical importance when dealing with sensitive information.

The overall landscape of awareness and implementation still exhibits significant gaps. This discrepancy points to a gap in the integration and application of these comprehensive measures in continental policies, underscoring the need for enhanced alignment and implementation to ensure that the global directives effectively trickle down and manifest in tangible, measurable improvements in health outcomes across all regions. A regional difference could be attributed to the awareness and implementation of digital health policies. Among these factors, cultural diversity plays a crucial role, since it influences healthcare perceptions and practices, affecting the acceptance and adoption of digital health technologies [ 37 , 38 ]. Economic inequalities across regions affect the accessibility and affordability of healthcare technologies, which in turn influence the ability to develop and implement comprehensive digital health policies. In addition, differences in technological infrastructure and internet penetration rates contribute to this divide [ 39 ], with regions with more advanced technological infrastructure being better equipped to implement digital health solutions. A comprehensive approach to advancing global health outcomes requires an understanding of these intricate regional dynamics.

Most notably, none of the policies included in this study explicitly outlined methods and data sources for SDoH data collection or specified types of SDoH data. A lack of clear strategies for data collection and an absence of data sources indicates that understanding the extent of health disparities and addressing them may prove challenging. Collecting and analyzing SDoH data and its impact on health are crucial, with a particular emphasis on promoting equity when implementing digital health strategies [ 40 , 41 ]. Prioritizing SDoH data collection is the first step to achieving holistic healthcare. Digital technologies like electronic health records, patient portals, and telehealth can play a foundational role in gathering and assessing SDoH data, facilitating information exchange between patients and interdisciplinary healthcare teams, and enabling community-level interventions to improve underlying social and economic conditions that contribute to health disparities [ 42 ]. Likewise, a diverse range of rapidly evolving mHealth tools, including apps, opens up new avenues for collecting patient-generated data, potentially including information on SDoH [ 43 , 44 ].

In addition, there are currently no established best practices for collecting SDoH data, and the absence of government mandates for SDoH data collection, as identified in our scan, may be attributed to this lack of established practices to include these considerations in digital health policies. A previous study examined data collection methods and SDoH utilization in general internal medicine wards [ 45 ]. The analysis encompassed only eight studies, which primarily collected SDoH data related to food security, housing, transportation, employment, education, income, disability, and social support; however, there were limitations in the lack of details regarding SDoH data collection methods. Hence, to facilitate systematic data collection, it is imperative to conduct psychometric testing of tools related to SDoH and integrate them into electronic health records. Further research is required to fill this identified gap in order to develop an international standard.

The increasing integration of digital technologies and applications in healthcare enables the provision of a wide range of healthcare services. However, digital transformation introduces unique challenges in health equity and SDoH. As reliance on these digital tools grows, there is a potential for disparities to expand, creating gaps between individuals with digital skills and access and those lacking such resources. To mitigate this challenge in digital health equity, policies related to health technology should establish standards to ensure that these digital technologies fulfill their intended purpose of enhancing access and improving care quality for a diverse population while preventing unintended consequences [ 46 , 47 ]. However, in our evaluation, policies from North America, Europe, Asia and Africa lacked strategies to mitigate digital divide in their policy documents. These policies should be established in a manner that guides healthcare systems, industries, and workers in anticipating, preparing, and suggesting potential strategies to overcome challenges during the implementation of digital technology in healthcare.

In particular, the importance of SDoH within the framework of precision prevention should be highlighted. Integrating SDoH into the precision prevention field allows healthcare providers to gain a comprehensive understanding of patient-specific risk factors, enabling more accurate risk assessments and tailored interventions [ 48 ]. Furthermore, the combination of precision prevention and the evaluation of SDoH holds significant potential to address and mitigate health inequities [ 49 , 50 ]. For example, this approach facilitates the proactive identification of individuals most vulnerable to health disparities through data-driven insights and predictive modeling, thereby allowing for tailored interventions, resource allocation, and policy implementation to address the root causes of health disparities. Thus, to establish a systematic and scalable approach for collecting, analyzing, and utilizing SDoH data, the integration of SDoH into digital health strategies is essential. Comprehensively understanding global digital health strategies and their standard policies regarding the incorporation of SDoH considerations ensures that effective precision prevention can be accomplished. Consequently, it promotes more equitable and effective healthcare outcomes by enabling tailored interventions and policies that address the root causes of health disparities across diverse populations.

### 4.1. Limitations

A notable limitation of our study is that it only includes policies implemented after the year 2020, which means that earlier policies were not considered for analysis. This temporal restriction may omit valuable insights from earlier policy developments in the field of digital health. Additionally, we excluded policies written in languages other than English or the co-authors' primary languages ( *i.e.* , Finnish and Korean). This language-based exclusion could potentially lead to the omission of policies from countries where English or the co-authors' languages are not widely spoken. Consequently, digital health policies may not be adequately represented in the study. Furthermore, some countries were unable to access their government websites, which limited our ability to retrieve and analyze their digital health policies. This access constraint might result in an incomplete representation of policies from certain regions. In certain countries or regions, SDoH may already be well-integrated into their healthcare systems and considered inherent in their strategies, thus not necessitating explicit specification. Lastly, the countries included in our study may not be fully representative of their respective continents. The selection of countries was influenced by factors such as accessibility of policy documents and available resources, which could introduce a bias in the continental representation of digital health policies.

### 4.2. Directions for the future research

Given the findings and limitations of our international environmental scan review, several recommendations for future research can be made. First, we recommend the establishment and refinement of regulatory frameworks as well as health policies that facilitate the integration of SDoH into the global healthcare system. This endeavor aims to provide policymakers with comprehensive insights into the extent to which digital health policies consider these determinants. A significant aspect of our study will guide these policymakers in effectively incorporating SDoH in digital health strategies. Furthermore, to ensure a truly global representation, more research is needed to extend digital health policies from a broader spectrum of countries, particularly emphasizing the inclusion of non-English strategies previously excluded due to language barriers. This expansion is vital for a more holistic global understanding and approach to digital health strategies. Also, the interdisciplinary nature of research, including social services, psychology, and political health studies, is essential in broadening the scope and depth of integration of SDoH in digital health. By embracing diverse perspectives and linguistic inclusivity, more policies will pave globally representative digital health policies.

## 5. Conclusions

Digital health strategies are in use globally, but their effective implementation faces challenges, necessitating each country's governments to continually introduce, evaluate, and adjust digital health policies. A comprehensive plan is vital to enhance the global introduction, implementation, and evaluation of digital technologies within economic and geopolitical networks. For future regulation to prioritize equitable and inclusive health technology, the consideration of SDoH within digital health policies (or policies related to digital determinants of health) should be thoroughly integrated into policy actions.
